# Homosexual men in HIV serodiscordant relationships: implications for HIV treatment as prevention research

**DOI:** 10.7448/IAS.18.1.19884

**Published:** 2015-05-25

**Authors:** Benjamin R Bavinton, Fengyi Jin, Limin Mao, Iryna Zablotska, Garrett P Prestage, Andrew E Grulich

**Affiliations:** 1 The Kirby Institute, University of New South Wales, Sydney, Australia; 2 Centre for Social Research in Health, University of New South Wales, Sydney, Australia; 3 Australian Research Centre in Sex, Health and Society, La Trobe University, Melbourne, Australia

**Keywords:** HIV treatment as prevention, men who have sex with men, homosexual men, serodiscordant couples, HIV viral load, HIV transmission

## Abstract

**Introduction:**

Studies in heterosexual HIV serodiscordant couples have provided critical evidence on the role of HIV treatments in reducing HIV transmission risk. However, there are limited data regarding the effect of treatment on HIV transmission in homosexual male couples. We examined features of male homosexual HIV serodiscordant relationships that may impact upon the design of HIV treatment and transmission studies.

**Methods:**

Data were from a prospective cohort study of HIV-negative homosexual men in Sydney, Australia. Men were followed up with six-monthly interviews and annual testing for HIV. Characteristics of men in HIV serodiscordant and seroconcordant relationships at baseline were compared, and a longitudinal analysis performed of rate of relationship break-up and of HIV incidence.

**Results:**

At baseline, 5.5% of participants (*n*=79) had an HIV-positive partner. Most (80.8%) of these relationships were non-monogamous, and 36.7% of men reported recent unprotected anal intercourse (UAI) with casual partners. The rate of relationship break-up was 29.5 per 100 person-years. Half of men in serodiscordant relationships (49.4%) reported recent UAI with their regular partners. HIV incidence was 2.2 per 100 person-years. It was substantially higher in relationships of less than one year's duration (6.1 per 100 person-years) and in men who reported unprotected receptive anal intercourse with ejaculation with their regular partners (15.5 per 100 person-years).

**Conclusions:**

Levels of HIV transmission risk and incidence were high, particularly in early relationships. Rates of relationship break-up were high. These data suggest that studies of HIV treatments and transmission in homosexual serodiscordant couples should focus on early relationships so as not to underestimate risk, and sample sizes must allow for high rates of relationship break-up.

## Introduction

Longitudinal studies in heterosexual HIV serodiscordant couples have been critical in delineating the role of the treatment of HIV-positive partners with antiretroviral therapy (ART) in reducing the risk of HIV transmission within these couples. Observational studies of such couples reported that HIV treatment of HIV-positive partners was associated with greatly reduced transmission [1]. In 2011, a randomized clinical trial, the *HIV Prevention Trials Network (HPTN) 052 Study*, confirmed that heterosexual couples randomized to immediate treatment of the HIV-positive partner had a 96% reduction in HIV transmission to the HIV-negative partner [2]. In contrast to heterosexuals, there has been very limited data from prospective studies of the relationship between HIV treatment and HIV transmission in male homosexual serodiscordant couples [3,4]. Given that the transmission probability of HIV via anal intercourse is around 10 times greater than for vaginal intercourse [5], the results from heterosexuals cannot be simply extrapolated. In addition, as anal sex is not uncommon among heterosexuals [6,7], results from studies in homosexual men may also have a broader relevance to the overall effectiveness of HIV “treatment as prevention” in the general population.

Internationally, while there has been some focus on the use of pre-exposure prophylaxis (PrEP) in homosexual men [8,9], only one study to date has reported findings on the impact of HIV treatment in HIV-positive partners on transmission to their HIV-negative partners in homosexual male serodiscordant couples: the *Partners of people on ART – a New Evaluation of the Risks* (*PARTNER*) *Study* in the United Kingdom and Europe [4]. The interim analysis presented in early 2014 provided promising but inconclusive results. Amongst 308 homosexual male serodiscordant couples, no phylogenetically linked HIV transmissions were observed in two years of follow-up. However, the results were statistically consistent with a possible risk of up to 1.17% per year for couples who reported any unprotected anal intercourse (UAI), and up to 1.97% per year in those couples where receptive UAI (with or without ejaculation) was reported [10].

Since HIV treatment of the HIV-positive partner has been proven to be highly effective in preventing HIV transmission to the HIV-negative partner in heterosexual serodiscordant couples, it is generally agreed that a similar randomized trial in homosexual male couples is not ethical [3,11]. Rather, longitudinal observational studies of HIV treatment and transmission are critical [12]. There are several key issues that may impact upon the HIV incidence in male homosexual serodiscordant couples and the feasibility of enrolling them into longitudinal studies. For example, higher levels of non-monogamy compared to heterosexuals, the relative longevity of relationships and whether serodiscordance plays any role in this, risk behaviour within couples and, in particular, the question of whether the HIV risk profile for HIV-negative partners changes over time. However, relatively little is known about the characteristics of these relationships and how they might impact upon such studies. In a cohort of HIV-negative homosexual men, we aimed to describe features of serodiscordant relationships, and to compare them to seroconcordant relationships, so as to consider key design features of prospective cohort studies of the role of antiretroviral treatment in reducing transmission in serodiscordant homosexual couples.

## Methods

### Participants

Participants were recruited into the *Health In Men Study* (*HIM*) from a wide range of community-based settings in Sydney, Australia between June 2001 and December 2004, and followed until 2007, as described in detail elsewhere [13]. Men were eligible if they met the following criteria: (1) reported having sex with other men within the previous five years; (2) lived in Sydney or participated regularly in its gay community; and (3) tested HIV-negative at baseline. Signed consent was obtained. The study was approved by the Human Research Ethics Committee at the University of New South Wales, Australia.

### Data collection

Participants underwent two interviews each year, reporting whether they had a primary regular sexual partner (defined by participants) currently and in the last six months, and the HIV serostatus of regular partners. Men who reported that they had an HIV-positive regular partner during the past six months were defined as being in a serodiscordant relationship. During follow-up, participants reported whether they were still in these same sexual relationships, if they had new regular partner/s or if they no longer had regular partner/s, as well as their perception of the HIV viral load of their HIV-positive partner (if applicable). Participants reported details of sexual behaviour with their regular partner/s and with other men, including the number of episodes of UAI separately for regular and for casual partners, for the insertive and receptive positions, and by HIV status of these partners (negative, positive or unknown). In addition, for receptive UAI, they reported episodes separately by whether or not ejaculation occurred inside their rectum. The reported types of UAI with the regular partner were regrouped for analysis according to their escalating risk of HIV infection, that is, no UAI; insertive UAI only; receptive UAI with withdrawal (men in this grouping could also report insertive UAI with their partner); and receptive UAI with ejaculation (men in this grouping could also report insertive UAI and receptive UAI with withdrawal with their partner). Men were tested for HIV antibodies annually.

### Statistical analysis

Statistical analyses were conducted using Stata (Version 12, Stata Corporation, College Station, Texas, USA). Characteristics of men in serodiscordant relationships and seroconcordant relationships at baseline were compared using logistic regression. The break-up rates of the serodiscordant relationships and seroconcordant relationships were compared in a longitudinal analysis, and predictors of relationship break-up for men in serodiscordant relationships were examined with Cox regression. HIV incidence per person-year was calculated in participants with HIV-positive primary partners and compared with men with HIV-negative primary partners [14]. Having such a partner was treated as a time-dependent co-variable. Men were included in this analysis if they reported a primary regular partner whose HIV status was known to them at any point during follow-up (*n*=1165), and excluded men who during follow-up never had a primary partner or had a primary partner whose HIV status the participant did not know (*n*=262). Cox regression was used to determine hazard ratios for categories of different HIV risk. For categories in which zero events were observed, incidence rate ratios and 95% confidence intervals were calculated using exact Poisson regression. Methods of ascertainment of HIV seroconversion in *HIM* have been described elsewhere [14]. All regression models used Type I error of 5%. For logistic and Cox regression models, the backward stepwise method was used. In all models, the *p*-value for overall trend was reported for continuous or ordinal variables, and *p*-value for heterogeneity was reported for categorical variables.

## Results

A total of 1427 HIV-negative participants were enrolled and the median age at enrolment was 35 years (range=18–75 years). The vast majority (95.2%) of participants self-identified as gay or homosexual. Follow-up interviews at one and two years were attended by 87 and 81% of participants, respectively, and the most common reason for loss-to-follow-up was relocation from Sydney (*n*=139, 9.74%). The cohort has been described in detail elsewhere [14,15].

### Characteristics of serodiscordant relationships at baseline

At baseline, 66.3% (*n*=946) of participants reported having a primary regular partner: of these, 8.4% (*n*=79) reported an HIV-positive primary partner; 70.6% (*n*=668) an HIV-negative primary partner; and 21.0% (*n*=199) had a primary partner of unknown HIV serostatus. Men in serodiscordant relationships were not different from men in seroconcordant relationships with regard to age (*p*-trend=0.246), primary partner's age (*p-*trend=0.368) or length of relationship (*p*-trend=0.156). The majority of men in serodiscordant and seroconcordant relationships had had sex with at least one other partner in the previous six months, and the proportion did not differ between the two groups (80.8 and 73.1%, respectively, *p*=0.145). Nearly three-quarters (72.2%, *n*=57) of men in serodiscordant relationships had been tested for HIV in the last six months, compared to half (50.7%, *n*=337) of men in seroconcordant relationships (*p*<0.001).

At baseline, more than half of the men in serodiscordant relationships (59.5%, *n*=47) and 76.4% (*n*=510) of the men in seroconcordant relationships reported UAI in the previous six months (*p*=0.001). UAI with regular partner(s) in the previous six months was reported by 49.4% (*n*=39) and 72.0% (*n*=481) of men in serodiscordant and seroconcordant relationships respectively (*p*<0.001). By contrast, UAI with casual partner(s) in the previous six months was reported by 36.7% (*n*=29) and 21.4% (*n*=143) of men in serodiscordant and seroconcordant relationships (*p*=0.003).

Men in serodiscordant relationships reported the viral load of their HIV-positive partner at baseline. While 39.2% (*n*=31) reported the partner's viral load to be undetectable (defined as under 500 copies per mL) and 26.6% (*n*=21) reported it to be detectable, one-third did not know the partner's viral load (34.2%, *n*=27). Participants who perceived that their partner's viral load was detectable or unknown were non-significantly less likely to report UAI with their partner in the previous six months (OR=0.45, 95% CI=0.18–1.1, *p*=0.091).

### Incidence and predictors of relationship break-up

Among the 747 HIV-negative men with known-HIV-status primary regular partners at baseline, the overall incidence of relationship break-up was 26.6 per 100 person-years. In men who reported being in a serodiscordant relationship at baseline, the incidence of break-up was 29.5 per 100 person-years (49 of 79 partnerships), and this was not statistically different from men in seroconcordant relationships (26.3 per 100 person-years; *p*=0.416). In men in serodiscordant relationships, those in longer relationships were less likely to break-up compared to men in relationships of less than six months at baseline (hazard ratio [HR]=0.39, 95% CI=0.18–0.84; Table 1). Men aged over 45 years at baseline were less likely to report relationship break-up (HR=0.20, 95% CI=0.06–0.67) than younger men, as were men who only engaged in insertive UAI in their relationship compared to men who had receptive UAI (HR=0.38, 95% CI=0.17–0.83).

**Table 1:** Predictors of relationship break-up in 79 homosexual men who reported being in an HIV serodiscordant relationship at baseline in the *Health in Men* study

	Relationship break-up					
	
	PY	*n*	Incidence (per 100 PY)	HR	95% CI	*p*
Length of relationship at baseline^a^						0.034
<6 months	18.84	10	53.09	1.0	–	
6–12 months	20.43	4	19.58	0.42	0.13–1.37	
1–2 years	20.05	8	39.89	0.75	0.30–1.91	
> 2 years	103.28	20	19.37	0.39	0.18–0.84	
Age at baseline^a^						0.010
< 35 years	53.82	22	40.88	1.0	–	
35–45 years	70.94	24	33.83	0.85	0.47–1.51	
> 45 years	41.39	3	7.25	0.20	0.06–0.67	
Partner's age at baseline^a^						0.006
> 5 years older	17.00	8	47.06	1.0	–	
Within 5 years	80.69	26	32.22	0.78	0.35–1.72	
>5 years younger	64.38	7	10.87	0.27	0.10–0.75	
UAI with regular partner/s at baseline^a^						0.371
No UAI	71.73	29	40.43	1.0	–	
Insertive only	58.93	8	13.58	0.38	0.17–0.83	
Any receptive	35.49	12	38.81	0.89	0.45–1.75	

a *p* for trend.

PY=person-years; HR=hazard ratio; 95% CI=95% confidence interval.

### HIV incidence in men in serodiscordant relationships

HIV incidence among men with known-HIV-status primary regular partners was examined in 1165 men over 3331.5 person-years (nearly two-thirds of the total study person-years of follow-up). Twenty-nine of these men seroconverted, and the overall HIV incidence was 0.87 per 100 person-years. Those who reported an HIV-positive primary partner had an HIV incidence of 2.20 per 100 person-years, compared to 0.71 per 100 person-years in those who had HIV-negative primary partners only (HR=3.12, 95% CI=1.38–7.05). HIV incidence was much higher in men who were in the first year of the serodiscordant relationship (6.1 per 100 person-years), and incidence decreased markedly in men reporting longer relationships (*p*-trend=0.003). In these partnerships, incidence was highest among men who reported at least one episode of receptive UAI with ejaculation with that regular partner (15.5 per 100 person-years, Table 2). Participants’ age and their perceptions of the viral load of their HIV-positive partners were not significantly related to incident HIV infection (*p*-trend=0.128 and 0.388, respectively).

**Table 2:** HIV incidence in men who reported an HIV-positive primary regular partner during follow-up

	HIV infections					
	
	PY	*n*	Incidence (per 100 PY)	HR	95% CI	*p*
UAI with regular partner^a^						0.001
Insertive UAI only	78.1	1	1.28	1	–	
Receptive UAI & withdrawal	49.3	2	4.05	3.11	0.28–34.6	
Receptive UAI & ejaculation	32.2	5	15.52	11.24	1.30–97.3	
No UAI	203.8	0	0.00	0.00^b^	0.00–14.9^c^	
Length of regular relationship^a^						0.033
<6 months	49.0	3	6.12	1	–	
6–12 months	32.9	2	6.07	1.37	0.22–8.68	
1–2 years	61.3	1	1.63	0.24	0.02–2.47	
>2 years	194.1	2	1.03	0.18	0.03–1.12	
Participant's age^a^						0.128
<35 years	106.7	5	4.69	1	–	
35–44 years	156.5	2	1.28	0.30	0.06–1.54	
>44 years	100.2	1	1.00	0.26	0.03–2.28	
Perceived partner viral load						0.388
Do not know	258.6	7	2.71	1	–	
Detectable	34.2	1	2.92	1.44	0.16–12.94	
Undetectable (<500 copies)	70.6	0	0.00	0.00^b^	0.00–2.54^c^	

a *p* for trend

b Incidence rate ratio (IRR)

c Poisson exact confidence interval of IRR.

PY=person-years; HR=hazard ratio; 95% CI=95% confidence interval.

## Discussion

About 5% of HIV-negative homosexual men enrolled in this community-based cohort in Sydney were in a serodiscordant relationship at study baseline, and half of these reported UAI with their regular partner(s) in the previous six months. Rates of relationship break-up were high, at 29.5 per 100 person-years, but this was not different to rates of relationship break-up in seroconcordant relationships. HIV incidence in serodiscordant relationships was 2.20 per 100 person-years, and was much higher in the first year of the relationship, and in men who reported receptive UAI in their relationship. Most men did not know their partner's viral load, and we documented no HIV seroconversions in those who reported that their partner had undetectable viral load.

Our data highlight several challenges in conducting prospective research on the role of ART in decreasing HIV transmission in homosexual male couples. Key issues include whether the prevalence of serodiscordant relationships is sufficient to achieve required sample sizes, the high rates of non-monogamy and break-up and how the HIV incidence in homosexual serodiscordant couples may affect sample size requirements.

In our community-based sample, 5.5% of HIV-negative homosexual men were in a HIV serodiscordant relationship (8.4% of men in relationships). This suggests that in the Australian state of New South Wales alone, there would be nearly 4000 HIV-negative men in serodiscordant relationships at any given time [16]. Thus, there should be more than enough eligible relationships to facilitate prospective studies of such couples, even in the presence of a high rate of relationship break-up.

Non-monogamy is an important concern for studies of HIV transmission in serodiscordant couples. In *HPTN 052*, approximately 5% of HIV-negative participants reported more than one partner in the previous three months. However, non-monogamy is likely to have been underreported in that study as only 71.8% of the incident infections were phylogenetically linked to the index HIV-positive partner [2]. In serodiscordant relationships in our study, in the past six months, sex with partners other than the regular partner was reported by 80.8%, and UAI with casual partners was reported by 36.7%. High rates of UAI outside of the primary relationship in homosexual couples have been reported in other studies [17–19]. These high levels of UAI imply that in a study on serodiscordant couples among homosexual men, it could not be assumed that the source of any infection was the index HIV-positive primary partner. As with recent serodiscordant couples studies in heterosexuals [4,20], phylogenetic testing would be vital to determine whether transmissions are linked [21].

Relationship break-up is a problem for any longitudinal study of couples [22]. In *HPTN 052*, around 3–5% of the couples in each arm ended their relationship during follow-up [2]. In our study, break-up rates among serodiscordant relationships were nearly 30% per year but were no different to rates in seroconcordant relationships. Given the importance of phylogenetic testing within couples where the HIV-negative partner seroconverts, consideration must be given to the potential challenges associated with collecting a final post-break-up blood specimen. Circumstances around break-up and seroconversion can be related, and break-up may follow as a consequence of high-risk episodes [23]. Inversely, break-up or conflict may also lead to risky practices with casual partners [24,25] and possibly also within relationships. In addition, our data indicated that shorter relationships had higher HIV incidence but were more likely to break-up during follow-up than longer relationships. This presents a challenge in study design. The newer relationships in which HIV transmission is most likely are also the relationships most likely to experience break-up. Thus, in recently formed couples, even a short period of follow-up is likely to be important, and our data suggest it would be important to target recruitment to newly formed couples in treatment as prevention cohorts.

The incidence of HIV of men in serodiscordant couples in our study was high (2.2 per 100 person-years), and was about 3-fold higher than in other men. The high incidence rate was unsurprising given the high levels of UAI, including receptive UAI, that were reported. For those who reported a serodiscordant relationship of one year or less, the HIV incidence was much higher (at around 6 per 100 person-years) than in those who reported longer relationships (around 1 per 100 person-years), demonstrating that the riskiest period for HIV-negative men in serodiscordant relationships is the first year of the relationship. This may be due to the relatively higher frequency of sex in newer relationships [26,27]. In addition, partners who stay HIV-negative after longer periods of time in serodiscordant relationships may have reduced genetic predisposition or acquired immunity through repeated exposure [22]. Men in new relationships need to be targeted to ensure that the true effect of HIV treatment in the HIV-positive partner on transmission can be measured. Restricting participation to “stable” couples who have mostly been together for a considerable period of time may have the effect of selecting a low HIV incidence population for study. Studies may benefit from targeting non-romantic yet regular sexual relationships (e.g. colloquially known as “fuckbuddy” relationships [28]). Many longer “committed” homosexual male relationships may begin in this way [29].

This study had the strength of being community-based with a high rate of participant retention, and is one of very few reported prospective studies of homosexual male serodiscordant relationships. Our analysis had several limitations. First, due to the *HIM* study's focus on HIV-negative individuals, the viral load of the HIV-positive partner was reported by the respondent and may have been inaccurate. Second, the data were collected when condom use was virtually the only HIV prevention method promoted to gay men, and certainly prior to any widespread discussion of HIV treatment as prevention or PrEP in the gay community. There is potential that the results of studies of ART-based prevention may have led to various changes in the attitudes and behaviour of men in homosexual serodiscordant couples. For example: explicit communication about viral load within couples may now be more common; viral load may be utilized to a greater extent in decision-making about condom use; and greater uptake of HIV treatment amongst HIV-positive men may impact upon the incidence rate within homosexual serodiscordant couples. Furthermore, although PrEP is not commercially available in Australia at the current time, PrEP demonstration projects have commenced in several cities. PrEP may affect studies on treatment as prevention: If HIV-negative partners take PrEP, the distinct risk reduction effects of treatment as prevention may become more difficult to assess. It is likely that larger sample sizes will be required, as well as careful monitoring of PrEP use throughout follow-up. However, despite these limitations, the analysis provides important insights regarding HIV-negative men in serodiscordant relationships and their implications for existing and future treatment as prevention research among gay men.

## Conclusions

Evidence regarding HIV treatment and transmission in homosexual male serodiscordant couples has been presented only on the interim analysis from the *PARTNER Study* in the United Kingdom and Europe [4,10], despite several studies of this kind in heterosexual couples. Determining the impact of HIV treatment and undetectable viral load on HIV transmission in homosexual male serodiscordant relationships is an urgent research priority. Along with *PARTNER*, we are aware of only one other ongoing study on treatment as prevention in homosexual couples: the *Opposites Attract Study*, being conducted in Australia, Brazil and Thailand [3,30]. The data from this analysis have highlighted important design features that should be included in future studies. The most important of these is that studies must make an effort to recruit homosexual men in newly formed sexual relationships, given the large reduction in HIV incidence observed after the first year of new relationships.
